# Machine Learning Prediction of Fall Risk in Older Adults Using Timed Up and Go Test Kinematics

**DOI:** 10.3390/s21103481

**Published:** 2021-05-17

**Authors:** Venous Roshdibenam, Gerald J. Jogerst, Nicholas R. Butler, Stephen Baek

**Affiliations:** 1 Department of Industrial and Systems Engineering, University of Iowa, Iowa City, IA 52242, USA; venous-roshdibenam@uiowa.edu; 2 Department of Family Medicine, University of Iowa, Iowa City, IA 52242, USA; gerald-jogerst@uiowa.edu (G.J.J.); nicholas-r-butler@uiowa.edu (N.R.B.)

**Keywords:** fall-risk detection, wearable shoe sensors, Timed-Up-and-Go test, convolutional neural networks

## Abstract

Falls among the elderly population cause detrimental physical, mental, financial problems and, in the worst case, death. The increasing number of people entering the higher risk age-range has increased clinicians’ attention to intervene. Clinical tools, e.g., the Timed Up and Go (TUG) test, have been created for aiding clinicians in fall-risk assessment. Often simple to evaluate, these assessments are subject to a clinician’s judgment. Wearable sensor data with machine learning algorithms were introduced as an alternative to precisely quantify ambulatory kinematics and predict prospective falls. However, they require a long-term evaluation of large samples of subjects’ locomotion and complex feature engineering of sensor kinematics. Therefore, it is critical to build an objective fall-risk detection model that can efficiently measure biometric risk factors with minimal costs. We built and studied a sensor data-driven convolutional neural network model to predict older adults’ fall-risk status with relatively high sensitivity to geriatrician’s expert assessment. The sample in this study is representative of older patients with multiple co-morbidity seen in daily medical practice. Three non-intrusive wearable sensors were used to measure participants’ gait kinematics during the TUG test. This data collection ensured convenient capture of various gait impairment aspects at different body locations.

## 1. Introduction

Falls are common in the older adult population, causing serious injuries [1]. U.S. Centers for Disease Control (CDC) statistics [2] show that the risk of death due to a fall begins to soar starting at the age of 65, with 27% of adults in the age range of 65–74 reporting one or more falls, increasing to 30% in those aged 75–84, and 37% of those 85 and older [3]. Additionally, the size of the population entering the higher fall-risk age is exponentially increasing [4]. Common injuries related to falls include hip and other bone fractures [5,6], as well as head injuries [6,7]. The injuries sustained from falls lead to emergency care treatment and hospitalizations [8]. Those who suffer a fall are further impacted by mental trauma [7], including fear of future falls, feelings of loss of independence, increased social isolation, and depression [1]. In addition to the suffering experienced by the individual involved in a fall, caregivers of that individual also face burden, increased fear, and stress [9,10]. Therefore, it is critical to recognize when an older adult has an increased risk of being involved in a fall in order to implement appropriate preventive measures to mitigate the risk.

A fall occurs when a person loses balance or consciousness caused by an outside trigger, a physical impairment, malnutrition, medication, or disease [11,12]. Fall risk factors are generally divided into intrinsic/physiological attributes and extrinsic/environmental risks [11]. Recognizing the presence of these risks through assessment is crucial in the prevention of future falls. Interventions include environmental alterations, gait and balance training, physical therapy, stopping or substituting different medications, etc. [13].

Some clinical tests such as the Four Square Step Test (FSST) [14], the Timed Up and Go (TUG) test [15], Functional Reach Test (FRT) [16], Step Test [17], and Berg Balance Scale (BBS) [18] have been designed to not only screen the balance, strength, stepping and gait of the older adult population but also to quantify functional mobility and analyze fall risk factors. Appendix A Table A1 provides an overview of each of these clinical tests. These assessments are accepted measures to evaluate different aspects of balance and mobility in the elderly population [14]. However, all of these measures lack a standardized approach with concise and straightforward instruction for both patients and providers. In 2013, the CDC developed the Stopping Elderly Accidents, Deaths and Injuries (STEADI) initiative to aid clinicians in assessing fall risk [19]. The STEADI initiative uses an algorithm to assess a patient’s fall risk in a clinical setting by a primary health care provider [19]. This algorithm accounts for the patient’s history and evaluates gait, strength, and balance, to identify possible contributing fall risk factors [19,20]. STEADI recommends several functional and clinical assessments such as the TUG test, 30-Second Chair Stand (30 Sec Stand) test, 4-Stage Balance test, and measuring orthostatic blood pressure [21,22] to screen for patients’ fall risk. STEADI also provides guidance on how to conduct the tests, recommends cut-off points for the measurements that indicate fall risk, and includes a Fall Risk Factors checklist which is completed by the patient [23].

Table 1 compares the clinical tests in terms of their efficiency in capturing the prominent gait and balance attributes indicative of fall risk. The features represented in Table 1 are the dominant criteria that the clinician considers in rating clinical fall-risk screening tests. Of the clinical tests mentioned in Table 1, the TUG test represents the most effective means for a practical fall-risk screening. TUG is the most efficient as far as the time required and patients’ ability to perform the test, while still evaluating 8 out of the 9 features thought to be important in assessing falls. The BBS test has a similar benefit to the TUG test to capture many mobility tasks crucial to detecting fall risk. However, it consists of 14 complex and time-consuming tasks. In contrast, the TUG test requires the patient to simply stand up from a chair, walk for three meters, turn, walk back to the chair, and sit down [22]. Thus, the TUG test has been widely adopted as a standard test to study balance and functional mobility and the associated issues such as falls [24,25].

Aside from the TUG screening test benefits, there is an evaluation deficiency in all the screening tests. For each clinical test, a dichotomous value of the quantified measurement (e.g., the TUG test’s time length) is suggested to determine if the person is at a risk of fall. This classification technique dichotomizes a continuous variable with a separation cut-off point, which could depend on a slight change of the selected cut-off point. Thus, the resulting classification using these clinical assessments is highly susceptible to bias. Moreover, performing and assessing all these tests and gathering all the patients’ medication and fall history is time-consuming and cumbersome. Overall, these methods are costly and prone to inaccuracies, relying heavily on patients to provide accurate data or on the clinician’s best judgment based on what they can see.

Researchers recently have begun using body-worn kinematic sensors to precisely quantify participant gait and balance attributes during the clinical tests [26,27,28]. The TUG test’s efficiency in capturing a wide range of kinematic movements has made it the most suitable clinical test to use to obtain kinematic sensor data. Body-wearable kinematics sensors were mounted on the subjects’ bodies to measure gait kinematics during the TUG test [26,27]. Machine learning techniques were applied to enhance the prediction with a multi-dimensional feature space of kinematics data.

Greene et al. [26] included a large sample of 349 community-dwelling older adults for the TUG test’s retrospective fall prediction. The study involved attaching two IMU wearable sensors to the participants’ anterior shanks. Twenty-nine parameters, including mean stride time, stance time, and step time, were determined to be statistically significant features in discriminating previous falls and were included in the fall-risk prediction. Their cross-validated logistic regression models obtained a mean sensitivity and specificity of 77.3% and 75.9%, respectively, and outperformed both TUG and BBS tests to predict prior fall incidents. Their contribution to the previous works was using wearable sensors to accurately measure the TUG test and extracting temporal gait features. However, their feature engineering could be time-consuming and demanding in terms of lab equipment and expertise.

Weiss et al. [27] had 41 participants perform the TUG test with a wearable accelerometer attached to their waist. They extracted accelerometer-driven parameters such as sit-to-stand and stand-to-sit times, amplitude range, average step duration, and gait speed. Multivariate logistic regression was used to predict the risk of falls, based on the history of falls, with resulting accuracy, sensitivity, and specificity of 87.7%, 91.3%, and 83.3%, respectively. Although they could achieve high accuracy, their study included small sample size and required feature engineering.

Buisseret et al. [28] addressed the feature engineering challenge using a convolutional neural network (CNN) on raw kinematics signals collected during a clinical 6 min walk test. The test was performed with an IMU sensor attached to the back of participants in the L4 lumbar position. They predicted the actual falls during six months after the test with relatively good accuracy, sensitivity and specificity of 75% and the prediction was improved compared with the traditional TUG test scores. Although gait and balance kinematics were measured over a longer period, the 6 min measurement did not include sitting and standing motions. The 6 min walk only includes walking and some turnarounds, and it requires the clinician to watch and walk the patient for the entire time.

All previous studies have used the actual falls before or after the gait measurement to predict fall risk. However, the fact that patients did not experience a fall does not necessarily indicate that they do not have a gait and balance impairment that might cause a fall. Past research studies on fall-risk predictions have based their conclusions on results where most tests were performed on a population that had met multiple inclusion or exclusion criteria. Their conclusions might not apply to the population typically seen in a primary care or geriatrics clinic. Additionally, gait feature extraction used in some prior studies requires domain knowledge of signal processing and gait physics, which is not necessarily the clinicians’ expertise. Machine learning algorithms such as CNNs can perform data-driven feature extraction; however, they require a large sample size to produce certain results. These issues are critical because clinicians look for fall-risk detection techniques that are simple and affordable in a clinical setting and can be conducted quickly by a primary doctor.

Although the prospective fall occurrence can be the best comparator when evaluating fall-risk assessment tools, acquiring such data requires a larger-scale longitudinal study. Training the prediction model based on the actual fall incidents for a short-term screening study could increase the risk of imposing a bias. For example, the individual might have a high risk of falling, but they have not yet experienced a fall. Conversely, the geriatrician’s assessment is based on the comprehensive guidelines defined by the CDC, which includes not only the history of falls but also the patient’s medications, prior diagnoses, and multiple gait and balance assessments. Often, geriatricians receive a referral from a primary physician to assess the fall risk of a patient, and they want to know the sensitivity of the assessment before recommending interventions. The goal is to provide interventions for people at risk of falls even if they have not yet fallen. Therefore, there is a pressing need for an efficient and precise data-driven fall-risk detection algorithm that can automate the cumbersome process of the clinical fall-risk assessment. Achieving this goal is subject to a comprehensive gait measurement, which is quick and straightforward. Furthermore, it is critical to evaluate the prediction robustness in recognition of patients with high fall-risk by evaluating the model’s sensitivity to the choice of a fall-risk probability cut-off point.

This study aims to enhance the geriatrician’s fall-risk screening test of the older adult population with the minimal and most uncomplicated means of measuring and evaluating risk factors. The goal is to provide a clinically practical fall-risk detection with comparable sensitivity to the geriatrician’s assessment that can be considered a replacement for the clinician’s time and effort. Fall-risk classification is performed only using the kinematic sensor data of the TUG test. While the TUG test is conducted on a subject as part of the STEADI recommendations, the subject’s gait acceleration and angular velocity signals are extracted using three IMU sensors mounted at three different locations on the body. This data collection is cheaper and more comfortable than long-term gait evaluation and results in less effort and stress for both patients and clinicians. A CNN algorithm predicts the risk of fall using the entire sensor signals; instead of deciding based on only one single variable, which depends on the choice of a cut-off point, the CNN model analyzes the comprehensive kinematics feature space. CNNs have the already built-in feature extraction process, which helps to digest the complex gait features. Eventually, our approach aims to help clinicians detect fall risk without going through the demanding process of applying the CDC protocols to evaluate patients’ intrinsic fall risk factors.

The contribution of the current work can be summarized as follows:

1. The geriatrician’s fall-risk assessment is facilitated by combining an affordable and convenient way of measuring patients’ gait and balance. This inexpensive method can provide performance comparable to the human clinician’s assessment.
2. This is the first paper to compare a prediction model with a geriatrician’s assessment of fall risk, which synthesizes information on fall risk factors (medical health status, gait impairments, and fall history), rather than only relying on the fall incidents, which can increase the error of false negatives.
3. Sensor location was navigated to guarantee data acquisition from three important body points that we consider relevant to fall-risk prediction. Comparison of kinematics data from three sensor locations is conducted to investigate the most effective measurement of risk factors.

## 2. Materials and Methods

RunScribe^TM^ (Scribe Labs, Inc., Half Moon Bay, CA, USA) [29] wearable IMU sensors were used to extract the subjects’ gait kinematics signals during the TUG test. A Machine Learning algorithm was implemented to predict the clinicians’ assessment of fall risk and the participants’ actual falls in the follow-up study.

### 2.1. Population

One hundred participants (51 males, 49 females), 65 years of age and older, who consented under Internal Review Board (IRB) guidance at the University of Iowa Hospitals and Clinics were evaluated with several gait and balance tests in the Geriatrics Clinic. Participants were composed of a diverse group of geriatric patients attending an academic geriatrics clinic. They included 10 with cognitive impairment, 9 with vestibular impairment or hearing aid use, 5 with past brain injuries, 4 with peripheral neuropathies, 3 with parkinsonism, and 20 with lower extremity orthopedic conditions. A board-certified geriatrician with 35 years of clinical experience (GJJ) performed multiple CDC standardized functional and medication assessments: the TUG, the 30 Sec Stand, and the 4-Stage Balance tests were administered, and the measurement of orthostatic blood pressure was obtained from the health record. Some subjects used walking-assistant equipment, such as a walker or cane, while performing the TUG test.

According to the clinician’s evaluation extensively reviewed by multiple geriatricians, the subjects were categorized into high risk of fall (fallers) and low risk of fall (non-fallers) using the functional test assessments (test scores and the clinician’s observation of movement disorders), number of medications, number of diagnoses, age, gender, BMI, and the Staying Independent Brochure (SIB) score. SIB is the subjects’ report of risk factors, including subjects’ history of falls. Fifty-four (23 males, 31 females) participants were classified as fallers. Table 2 provides a general description of the subjects’ characteristics and functional test scores. Among the physiological attributes, only age is significantly different between fallers and non-fallers (*p*-value = 0.004). The odds ratio shows that older ages are prone to a higher risk of fall. Medical record data showed the highest associated with being classified as a faller to be the number of diagnoses and the number of medications used (*p*-value < 0.001). All STEADI tools had a high odds ratio with a wide confidence interval (CI) associated with the geriatrician’s assessment of the participant being a faller (*p*-value < 0.001). The participants’ report of fall risk factors obtained with SIB scores had the highest odds ratio and widest CI. Although they showed a high association with the geriatrician’s assessment of falling in our case study, the wide CI reveals extreme uncertainty due to the small sample size.

**Figure 1:**
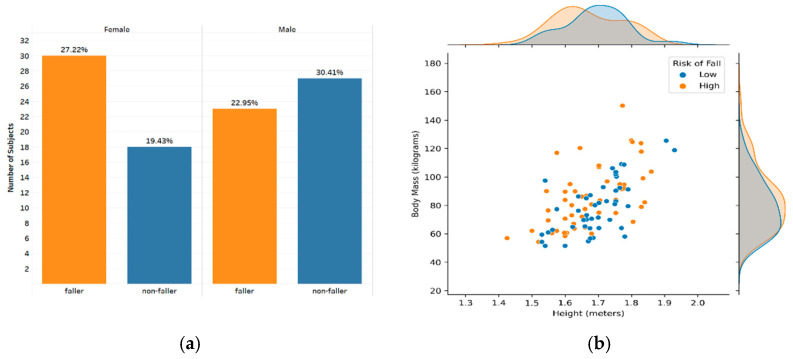
Descriptive summary of geriatrician’s fall classification versus subjects’ gender, height, and weight. (**a**) Represents the percentage of fallers and non-fallers in each group of females and males separately; (**b**) shows the joint distribution of fallers and non-fallers with respect to subjects’ height and body mass.

RunScribe^TM^ IMU pods were mounted on patients’ bodies at three locations before performing the clinical tests. Two pods were fastened tightly on the lace of the right and left shoe near the midfoot, while the third pod was attached to the collar of the subject’s clothing at the back of the neck. These sensor pods are respectively referred to as the right foot, left foot, and neck. After data collection, it was noted that one subject had missing data from the right foot sensor, and one subject from the left foot sensor. Therefore, we continued the study with the remaining ninety-eight participants (50 males, 48 females). Fifty-three (23 males, 30 females) out of 98 subjects were classified as fallers (with a high risk of fall), and the other forty-five (27 males, 18 females) were classified as non-fallers (with a low risk of fall). The summary of the distribution of geriatrician’s fall classification versus gender, height, and weight of the studied sample is illustrated in Figure 1. The acceleration and angular velocity signals collected from the sensors alongside the geriatrician’s evaluation of the subject as a potential faller or non-faller are used to study patients’ gait and balance.

### 2.2. Data Acquisition

**Figure 2:**
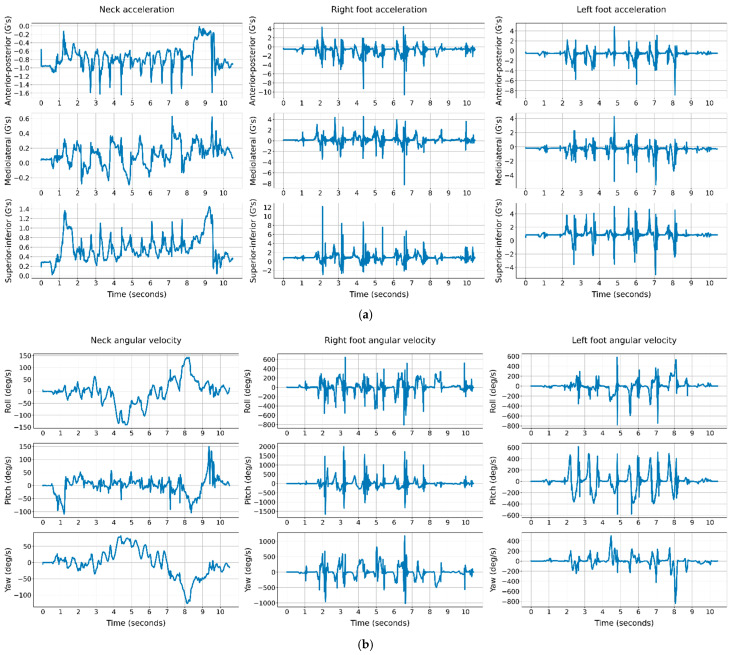
The neck, right and left foot kinematics signals of a subjects’ TUG test over time. (**a**) Acceleration signals; (**b**) angular velocity signals.

The IMU sensors captured data during the TUG test. Subjects were asked to stand up from a sitting position in a chair and walk at their usual pace for three meters, turn and walk back and sit again. The RunScribe^TM^ pods use MPU-9255 Micro-Electro-Mechanical Systems (MEMS), which contains a triple-axis accelerometer and a triple-axis gyroscope. The pods setting for these MEMS was to collect 3D kinematics data at a sampling rate of 250 Hz, within the range of 16 G acceleration and 2000 degree/s angular velocity, where G is the gravitational unit. The raw sensor data in this study contain the anterior–posterior (AP), mediolateral (ML), and superior–inferior (SI) acceleration and roll, pitch, and yaw angular velocity signals during the TUG test. The original signals of 250 Hz are sensitive to the abrupt magnitude drops and increases in a signal due to noise. Signal preprocessing is crucial to improve the original kinematics signals by filtering out very low and high frequencies out of the natural domain of human gait frequency. In this study, the signals were low-pass filtered 100 Hz using the Fast Fourier technique, and it was implemented by the resample function in Python’s SciPy library. Figure 2 illustrates the neck, right and left foot signals of a 71-year-old male subject, assessed with a high risk of fall.

Data normalization was conducted to convert all the input attributes into the same standard scale to help the machine learning model converge to the optimal solution. The acceleration and angular velocity signals were mapped separately from their respective range to [0, 1] using the minimum and maximum magnitude of acceleration and angular velocity signals across all subjects. Zero-padding was used to have all the input signals in the same size. This means that the time length of a subject’s signals is increased by adding zeros to the end of the signals until all the subjects have the same length as the longest TUG test’s length. Before feeding the raw signals into a deep learning model, signal segmentation was used to enhance the performance of CNNs. Motion signals were cut into three-second segments using a sliding window approach. A three-second window slides over a signal with a one-second stride and creates the three-second segments until the sliding window covers the entire signal. Every individual participant’s segmented signals were stacked channel-wise (3 acceleration and 3 angular velocity channels). The three-channel signal segments of each sensor location were considered as the input to the prediction models.

### 2.3. CNN Model with the Segmented Raw Signals of the TUG Test

**Figure 3:**
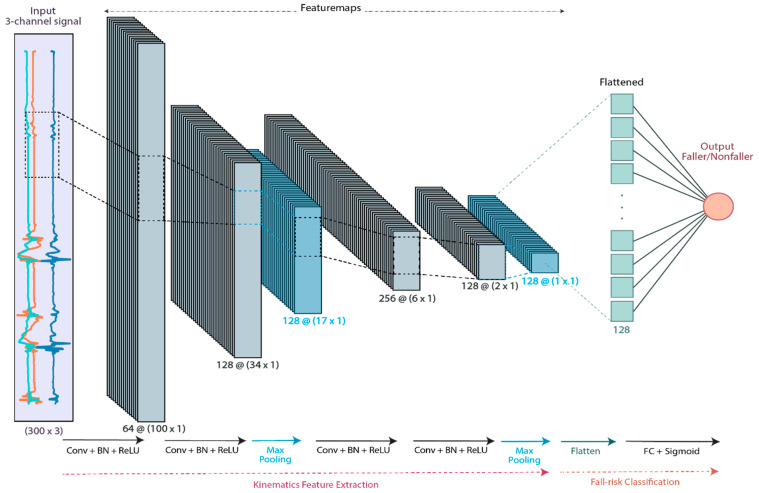
The CNN architecture of the proposed fall-risk classification model. The 3-channel acceleration or angular velocity 3 s segments are fed into the convolutional building blocks, and the high-level kinematics feature map is extracted. The features are flattened and classified as faller/non-faller by a fully connected neural network.

The tasks performed by our CNN algorithm were twofold: learning feature representation and fall-risk classification. The normalized signal segments were fed into the CNN as the model input. The overall geriatrician’s fall classification of the subjects was binarized into faller (output = 0) and non-faller (output = 1), representing the probability of fall. The CNN model consisted of 4 building blocks of 1-D convolutional (Conv) layers, each followed by a Batch Normalization (BN) and ReLU activation, which all together extracted the signals’ high-level gait features. Additionally, Max pooling layers were used after the second and the fourth ReLU activations to downsample the similar local information into a concentrated output. The feature maps of the last ReLU activation were flattened into a 1D array and then fed into a fully connected (FC) layer with a sigmoid activation function to serve as the predictor of the fall-risk probability. Then, binary classification of fallers versus non-fallers was performed using the threshold probability of 0.5. Figure 3 illustrates the architecture of the designed CNN model.

The participants were divided into training and test subjects with an 80% and 20% ratio. The proportion of fallers and non-faller was kept 50 to 50 in both training and test sets of patients. Each of these sets was randomly bootstrap resampled with a replacement for 100 iterations. In each iteration of bootstrapping, each set’s participants were resampled with replacement randomly for 100× number of subjects in the set. Therefore, we increased the sample size such that one training subject could exist more than once in the training set. Finally, all the raw signal segments of the training subjects built the training set, and all the raw signal segments of the test subjects built the test set. The CNN model was then trained using the training segments and then evaluated on the test segments. This process was repeated for 100 iterations. Eventually, the mean and 95% CI of classification accuracy (Acc), sensitivity (Se), specificity (Sp), F1-score, and the area under the Receiver Operating Characteristic curve (AUC) of the bagging CNNs are used to evaluate the classification performance.

## 3. Results

### 3.1. The Clinical Scoring Tests in Predicting Geriatrician’s Fall Classification

For each clinical assessment test, the dichotomous binary fall classification of the subjects was performed such that all the participants were in each test set. Each clinical test result was considered the predicted fall status, the overall geriatrician’s fall-risk assessment was assumed as the true fall-status, and the classification confusion matrix was built. Table 3 represents the classification results of each of the clinical tests.

A Receiver Operating Characteristic (ROC) analysis was performed for each clinical classification. J = Se + Sp − 1 (Youden’s J index) was also calculated for different cut-off thresholds to find the optimal threshold that maximizes J index (the trade-off between the true positive and false positive rates). As shown in Table 3, the 4-stage balance was the most sensitive among the practiced functional tests to detect fallers (70.37% sensitivity) with 81% accuracy and 0.64 J index. However, the optimal cut-off point of the 4-stage balance, similar to 30 sec stand cut-off points, differed from the respective cut-off points suggested by the CDC or used by the clinician. On the other hand, 14 s cut-off value, selected by the clinician and suggested by the CDC, was the optimal threshold for the TUG test with prediction accuracy, sensitivity, and specificity of 71%, 55%, and 89%.

Despite higher J index in the 4-stage balance test, we chose the TUG test for further sensor measurement because of its other benefits over the other tests. First, the optimal prediction achievable with the TUG test agrees with the clinician’s evaluation of the TUG test. Our goal was to find an approach that can eventually be a suitable replacement for clinician prediction. Second, due to the comparison in Table 1, in contrast to the 4-stage balance test, the TUG test implementation was simpler and incorporated multiple mobilities, resulting in a more precise kinematics measurement of gait and balance impairments associated with fall risk. Comparing these clinical test results with previous works could not be valid because they have applied different cut-offs [30,31] due to the researcher’s judgment or possibly adjusting the specific attributes in their studied population [15].

### 3.2. The CNN Prediction of Geriatrician’s Fall Classification

The bagging CNN algorithm was performed for each individual sensor location, one time using the 3-channel angular velocity signals, another time using the 3-channel acceleration signals. The prediction results of the six separate training experiments of the CNN model are reported with classification metrics in Table 4. The same bagging algorithm was also used with a support vector machine (SVM) model to evaluate the particular benefit of CNN. In Table 4, the notation gyro denotes that the ML model was trained with 3-channel gyroscope signals, and the notation accel denotes that the ML model was trained with 3-channel accelerometer signals. The SVM models were trained with the mean, standard deviation, and coefficient of variation of the three directional signals such that in each experiment, nine statistical variables were the inputs rather than the three-channeled time series that were fed into the CNN models.

Finally, the bagging ML models were compared with 100 iterations of bootstrap resampling of the traditional TUG test prediction. Contrary to the CNN models, the TUG test classification did not require training. In each iteration of bootstrap resampling, only twenty out of ninety-eight (10 fallers, 10 non-fallers) subjects were randomly selected. Then, a random sample was drawn with replacement from the selected subjects for 2000 (100 × 20) times to create the test set.

Table 4 contains the prediction results of the test set such that for each model, the classification metrics are reported with the mean and 95% CI of 100 bootstraps resampling. The AUC of an ML model shows a single model’s power to classify the fallers and non-fallers given the ROC analyzing the sensitivity and specificity trade-off with changing the fall-risk probability threshold. The p-value and CI for concordance (C-statistic) are based on the null hypothesis that assumes a model is a random guess (AUC = 0.5) versus the alternative that assumes the model can distinguish fallers and non-fallers (AUC > 0.5).

All kinematics-based ML models demonstrate an improvement of at least 19% higher sensitivity (neck CNN_accel), 4% higher F1-score (left CNN_accel) over the traditional TUG test. Although the clinical TUG test has 88% specificity with 72% AUC, it has very poor fall-risk detection (56% sensitivity). Among the three sensors, neck kinematics improved the performance of the ML models with a better trade-off between sensitivity and specificity and a higher power level to discriminate between fallers and non-fallers (*p*-value < 0.001 and AUC > 0.70). Overall, neck gyroscope signals boosted ML models to detect fall risk with higher sensitivity and AUC.

The Right and Left SVM models show very high sensitivity (>90%) with the cost of very low specificity (<5%) and lower AUC than the CNN models. The very low specificity and accuracy close to 50% demonstrate that these models do not learn any data patterns to detect the risk of fall, and they just classify most of the subjects as fallers. However, neck SVM models with high sensitivity have better performance in terms of accuracy and specificity. Although neck CNN_gyro has a 6% and 1% lower average sensitivity and F1-score than neck SVM_gyro, it is a more robust model on average in distinguishing fallers and non-fallers (5% higher AUC with shorter CI). The AUC of neck CNN_gyro indicates that, on average, a faller had a higher risk of falling than 76% of the non-fallers.

**Figure 4:**
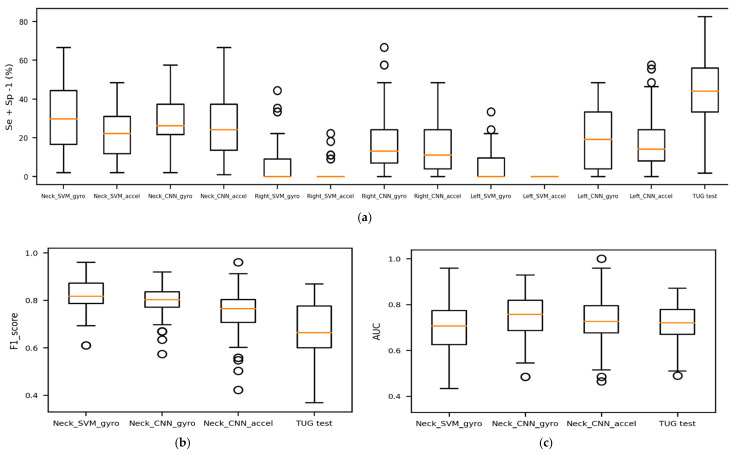
Comparison of models’ prediction performance. (**a**) Represents the models’ trade-off between sensitivity and specificity; (**b**) compares the best models’ F1-score with the traditional TUG; (**c**) illustrates the overall power of faller/non-faller discrimination for the best models and the traditional TUG.

Overall, the wide 95% bootstrap CIs of classification metrics show that the prediction’s significant uncertainty. Despite the data augmentation (bootstrap resampling and signal segmentation), the results still depended on which participants were selected in the training set. Figure 4 compares the J index, F1-score, and AUC of the models.

### 3.3. The CNN Prediction of the Follow-Up Falls Report

We conducted a follow-up survey of the subjects 6 to 12 months after the geriatrician’s assessment. They were asked if they experienced any falls since their TUG test in the clinic. Among 98 subjects, 87 (43 females, 44 males) responded to the follow-up question. Twenty-five (11 females, 14 males) reported falls (classified as fallers), and 62 (32 females, 30 males) reported no falls (classified as non-fallers). The reported fall status was not consistent with the geriatrician’s classification. Results in Table 5 show that the bagging CNN models cannot accurately predict the patients’ actual falls (very low average sensitivity with wide CI), and the SVM models have a very weak performance (sensitivity close to 0). The ML model used the gait kinematics over a short amount of time, collected more than six months before the actual falls. Therefore, the sensor data and the fall incidents are not closely associated. In addition, the imbalanced number of fallers and non-fallers (28% fallers) in the follow-up report could cause significantly higher specificity than sensitivity for the screening TUG test. A very low AUC shows that even adjusting the fall-risk probability threshold does not improve the prediction performance (*p*-value > 0.05 and/or average AUC very close to 0.50).

### 3.4. The Geriatrician’s Classification of the Follow-Up Falls Report

We used the geriatrician’s fall assessment to predict the follow-up falls report. The geriatrician’s classification labels were considered the predicted values and the follow-up falls report as the actual falls. The geriatrician’s fall assessment could predict the actual future falls with the accuracy, sensitivity, specificity, and F-1 score of 61%, 76%, 55%, and 0.53. Twenty-eight participants predicted as fallers reported no falls (false positives), and 6 of the participants predicted as non-fallers reported at least one fall (false negatives). Compared to the CNN prediction of actual falls, the geriatrician’s fall classification was a better predictor of the follow-up actual falls because the geriatrician assessment included multiple additional risk factors such as patients’ medications and diagnoses that could include long-term risk factors.

## 4. Discussion

The use of small non-intrusive IMU sensors attached to a subject provided an inexpensive alternative approach for the classification of a subject’s fall-risk status. Using only the participants’ TUG test kinematics instead of using the 6 min walk test kinematics and the traditional TUG test, as performed by Buisseret [28], our method classified subjects with at least similar sensitivity as an experienced clinician and would have required less clinician time. Using the single TUG test could reduce the risk and effort of having patients perform additional tests.

To avoid loss of generality imposed by sample bias, we used multiple techniques. Our study population consisted of 98 subjects with various physiological attributes and different health conditions such as cognitive and physical impairments. This contrasts with the other studies where co-morbidities were either disregarded, or in the case of Green et al., who used a largest sample size of 349 participants, participants with major cognitive or physical disorders were excluded. By including patients with impairments, we were able to have a sample that was consistent with the typical population seen in the geriatric clinical setting. To further avoid loss of generality, we estimated the prediction uncertainty due to sample selection. Each ML model was trained on a random selection of 80% of participants, and testing was performed on the remaining 20%. This random selection repeated in every 100 iterations of our bagging model added generality to our prediction and decreased the risk of overfitting to the selected subjects. At each bootstrap iteration, the subjects in each training and test set were resampled with a replacement for 100 times of each set’s sizes. Then, each subject’s signals were segmented into three-second window segments.

To our knowledge, this study is the first that predicts the outcome of a geriatrician’s fall-risk screening test rather than the actual previous or future falls predicted in prior studies (e.g., [26,27,28]). Using the CNN method with neck angular velocity, we achieved a high sensitivity of 86% compared to only 56% sensitivity using the traditional TUG test. However, the specificity result was 41%, below what is preferred in a diagnostic test to rule in a disease process [32,33], such as the hip fracture resulting from a fall. As the purpose of our study is a screening test and not a diagnostic test, the low specificity was not considered to be significant, while sensitivity was in a range to support better screening to rule out the high risk of fall [32,33]. However, the high false positives due to the resulting specificity can lead to unnecessary fear of falling among the subjects. To address the increased risks associated with fear of falling, the clinicians investigated this explicitly in this study’s SIB questionnaire. If a patient is screened as possibly high risk for falls, further interventions such as medication reduction, physical therapy, and exercise programs are recommended, which help alleviate the fear of falling.

The SVM and CNN models are compared based on two factors, sensitivity versus specificity, and AUC, where the model prediction has low responsiveness to the fall-risk probability cut-off at the final stage of classification. Although the neck CNN_gyro has lower sensitivity than the neck SVM_gyro, its higher AUC shows that it is more robust in distinguishing fallers from non-fallers while changing the fall-risk probability threshold.

As CNN analyzes the entire signal and performs data-driven feature extraction, there is no risk of missing any information by only introducing the statistics of the signals as was demonstrated with the SVM model in this study. Therefore, the neck CNN_gyro shows higher potential than the neck SVM_gyro for better performance with a more accurate sensor data acquisition system.

The gait feature engineering process used by Greene et al. [26] and Weiss et al. [27] focused on sensor-driven parameters such as temporal gait features to build prediction algorithms. However, relying only on kinematics signals to detect gait events’ timing can be prone to human error in selected filtering or rule-based algorithms. Therefore, their feature engineering required validation against a reference motion capture system to assure accurate extraction of gait features. Overall, gait feature engineering requires a certain amount of expertise in signal processing and gait analysis, given accurate sensor data acquisition. A combinatory sensor system to assure the synchronized left and right foot signals could be demanding and impractical for a low-cost screening at clinics. Our solution used CNN models with raw kinematics signals to perform data-driven feature extraction and faller classification. This automated feature representation reduced the costly feature extraction process by saving the required time and expertise effort. In addition to the kinematics data acquisition, the CNNs’ performance was improved by sample augmentation, signal resampling curation, and signal segmentation.

While CNN can automate feature engineering and eliminate the bias of human error and costs of motion capture lab facilities to validate manual feature engineering, its learning process remains unclear. In future studies, Grad-CAM visualization [34] could be used to localize the segments in the kinematics signals indicative of a high risk of fall, assisting clinicians in discovering the gait attributes that are closely associated with a high risk of fall.

The IMU sensors were placed on the participants’ neck, right shoe, and left shoe to investigate the importance of sensor location in fall risk prediction and evaluate the locational kinematics risk factors. The placement of the sensors’ effect on prediction was evaluated by training the CNN models separately for angular velocity and acceleration, as well as separately for right foot, left foot, and neck. Previous studies placed the sensor in the body center’s proximity (e.g., waist) to approximate the body centroid motion [28,35]. Other studies that aimed to analyze the gait features closely installed the sensors closer to the feet [36,37,38]. In our study, the neck sensor provided the TUG test kinematics associated with the upper torso and outperformed the foot sensors in fall-risk prediction. With proper synchronization, the foot sensors may have been more useful for engineering gait features.

Sensor synchronization was a limiting factor in this study. Due to the lack of automatic synchronization between sensors, each sensor relied on its own internal clock to keep time, and the resulting sensor data were collected in varying non-uniform time. The RunScribe sensors provide a method for calibration to prevent this; however, it would have required each participant to collect sample data by running for an extended distance before a test. This was unfeasible with our subjects. This timing issue hindered the ML implementation when combining separate sensors and limited a valid computation of temporal and spatial gait features. Some researchers have worked to solve this problem through the use of highly customized wirelessly networked IMUs or through the use of extensive camera systems connected to real-time references to guarantee accurate kinematics data collection for different purposes [39,40]. These studies do provide more reliable results than our study, but at a much greater cost of time and resources, and restrictions in deploying the systems to the general clinical setting.

Another limitation discovered in the results of the CNN classification in our follow-up fall reports revealed that AUC and sensitivity drastically deteriorated. This poor performance could be attributed to imbalanced follow-up data or the use of late fall incidents as the “ground truth” fall status for the TUG test. An actual fall incident that occurred more than six months after the TUG test could not be closely associated with fall risk factors captured at the time of the initial tests. The balance and mobility strength of older adults can change drastically over that time period. Other studies attempted to account for this by contacting participants monthly for fall reports [28]. In this situation, the geriatrician’s fall classification was a better predictor of the follow-up falls because it includes some long-term aspects of the subjects’ ambulatory, locomotion, and health condition. The geriatrician’s fall classification investigates fall risk factors using patients’ fall history, functional test scores, medications, and diagnoses, while the CNN classification only relies on the short TUG test kinematics at the time of testing.

## 5. Conclusions

Fall prediction in the older adult clinic population is an important and demanding task for geriatricians. It is limited to capturing only intrinsic risk factors for falling and, therefore, is likely not as accurate as a system that includes extrinsic risk factors found in the patient’s home environment. We proposed a fall-risk prediction model that can detect a geriatrician’s fall-risk assessment using cheap wearable sensors. The study was performed in a real-world clinical environment using simple procedures to obtain results. The study has demonstrated the use of machine learning techniques applied to sensor data obtained during the TUG test that can closely align with the experienced geriatrician’s ability to predict falls. Such machine learning techniques may become valuable clinical tools that could assist less experienced clinicians in being as accurate as an experienced evaluator of intrinsic fall risk factors. The final goal of this area of research is to create a non-intrusive sensor system that can measure intrinsic risk factors while the patient is interacting with the home environment.

## Figures and Tables

**Table 1:** Comparison of functional clinical fall-risk screening tests to find the simplest test with the most beneficial risk-factor measurement. +/− denotes if a specific criterion is met in a clinical test. The total row counts the number of existing features for each test.

Features	Clinical Tools						
	FSST	Step Test	TUG	FRT	BBS	4-Stage Balance	30 Sec Stand
Time required <a couple of minutes	+	+	+	+	−	+	+
Ease of performing	−	−	+	+	−	−	−
Measures static stability	−	−	+	+	+	+	−
Measures dynamic stability	+	+	+	−	+	−	+
Gait motion	−	−	+	−	−	−	−
Turning motion	−	−	+	−	+	−	−
Sitting and Standing motions	−	−	+	−	+	−	+
Reaching forward	−	−	−	+	+	−	−
Stepping	+	+	+	−	−	−	−
Total	3	3	8	4	5	2	3

**Table 2:** Summary statistics of participants’ attributes, medications, health and fall history, and fall-risk assessment measurement scores.

Summary Statistics	Age (Years)	Gender (Female vs. Male)	BMI (kg/m^2^)	# of Diagnoses	# of Movement Disorders	# of Medications	# of Psychoactive Medications	TUG (14 s or > vs. <14 s)	4-Stage Balance (30 s or < vs. >30 s)	30 Sec Stand (8 or < vs. >8 Stands)	SIB Score (4 or > vs. 0–3)
Mean (range)	75.41 (65–96)	49%	28.8 (18.30–47.74)	8.43 (1–19)	0.47 (0–4)	7.70 (0–21)	0.91 (0–5)	14.1 (7–98)	31.4 (4–40)	10.55 (0–23)	3.24 (0–12)
Odds ratio of being a faller (95% CI)	1.09 (1.03–1.16)	2.10 (0.94–4.67)	1.08 (1.00–1.16)	1.44 (1.23–1.69)	2.56 (1.21–5.39)	1.34 (1.17–1.55)	1.66 (1.13–2.44)	10.25 (3.51–29.96)	28.66 (7.81–105.71)	14.33 (3.96–51.87)	44.00 (9.57–202.35)
*p*-value	0.004	0.070	0.042	<0.001	0.014	<0.001	0.009	<0.001	<0.001	<0.001	<0.001

**Table 3:** Functional tests’ classification results with the geriatrician’s cut-off points.

Fall-Risk Assessment Tools (Fallers vs. Non-Fallers)	Acc (%)	Se (%)	Sp (%)	AUC	J Index	Optimal Cut-Off
TUG (14 s or > vs. <14 s)	71.00	55.55	89.13	0.72	0.45	14
4-stage balance (30 s or < vs. >30 s)	81.00	70.37	93.48	0.82	0.64	32
30 sec stand (8 or < vs. >8 stands)	70.00	50.00	93.47	0.71	0.43	10

**Table 4:** Fall-risk classification of geriatrician’s fall assessment using ML with the kinematics measures of the TUG test and comparison with traditional clinical TUG test.

Sensor	Classification Method	Acc (%)	Se (%)	Sp (%)	J Index	F1-Score	AUC	C-Statistic (95% CI)	C-Statistic *p*-Value
-	Clinical TUG test	70.65 (53.80, 85.78)	56.02 (27.48, 81.64)	88.53 (67.70, 100)	0.44 (0.10, 0.73)	0.67 (0.41, 0.85)	0.72 (0.55, 0.87)	25.70 (0.71, 0.74)	<0.001
Neck	SVM_gyro	67.13 (50.00, 80.00)	92.51 (72.72, 100)	36.11 (11.11, 66.67)	0.29 (0.04, 0.57)	0.81 (0.69, 0.92)	0.70 (0.51, 0.90)	18.57 (0.68, 0.73)	<0.001
	SVM_accel	62.39 (46.87, 75.00)	83.14 (54.54, 100)	36.57 (4.17, 66.67)	0.21 (0.02, 0.46)	0.77 (0.62, 0.88)	0.71 (0.51, 0.87)	23.05 (0.69, 0.73)	<0.001
	CNN_gyro	66.21 (50.00, 80.00)	86.51 (56.82, 100)	41.27 (11.11, 66.67)	0.28 (0.05, 0.57)	0.80 (0.67, 0.92)	0.75 (0.54, 0.92)	25.20 (0.73, 0.77)	<0.001
	CNN_accel	63.08 (50.00, 75.00)	75.47 (45.45, 100)	47.93 (22.22, 66.67)	0.25 (0.01, 0.48)	0.75 (0.55, 0.88)	0.73 (0.49, 0.89)	20.32 (0.71, 0.75)	<0.001
Right	SVM_gyro	56.06 (50.00, 67.87)	98.00 (81.82, 100)	4.89 (0.00, 33.33)	0.05 (0.00, 0.33)	0.76 (0.71, 0.84)	0.52 (0.41, 0.71)	3.33 (0.51, 0.53)	<0.001
	SVM_accel	55.35 (55.00, 60.00)	99.64 (95.22, 100)	1.22 (0.00, 11.11)	0.01 (0.00, 0.11)	0.76 (0.73, 0.79)	0.50 (0.43, 0.55)	1.55 (0.49, 0.51)	0.061
	CNN_gyro	59.77 (45.00, 79.50)	83.18 (46.82, 100)	31.04 (0.00, 66.67)	0.17 (0.00, 0.56)	0.75 (0.59, 0.91)	0.66 (0.47, 0.84)	14.82 (0.64, 0.68)	<0.001
	CNN_accel	58.33 (45.00, 75.00)	79.57 (36.36, 100)	32.37 (0.00, 66.67)	0.16 (0.00, 0.44)	0.72 (0.50, 0.86)	0.61 (0.38, 0.81)	9.92 (0.58, 0.63)	<0.001
Left	SVM_gyro	56.65 (55.00, 65.00)	99.36 (90.91, 100)	4.55 (0.00, 22.22)	0.04 (0.00, 0.22)	0.77 (0.73, 0.82)	0.53 (0.43, 0.66)	4.77 (0.52, 0.54)	<0.001
	SVM_accel	55.00 (55.00, 55.00)	100 (100, 100)	0.00 (0.00, 0.00)	0.00 (0.00, 0.00)	0.76 (0.76, 0.76)	0.50 (0.50, 0.50)	−1.00 (0.49, 0.50)	0.841
	CNN_gyro	60.91 (45.00, 73.50)	81.13 (39.09, 100)	36.20 (0.00, 66.67)	0.19 (0.00, 0.45)	0.75 (0.51, 0.86)	0.68 (0.48, 0.88)	15.71 (0.65, 0.70)	<0.001
	CNN_accel	59.41 (40.00, 78.50)	82.89 (36.36, 100)	30.70 (0.00, 66.67)	0.18 (0.00, 0.53)	0.71 (0.00, 0.90)	0.63 (0.37, 0.84)	10.45 (0.61, 0.65)	<0.001

**Table 5:** The ML prediction of follow-up fall incidents for each sensor location using the TUG tests’ kinematics signals.

Sensor	Classification Method	Acc (%)	Se (%)	Sp (%)	J Index	F1-Score	AUC	C-Statistic (95% CI)	C-Statistic *p*-Value
Neck	SVM_gyro	70.00 (58.19, 72.22)	2.20 (0.00, 20.00)	96.08 (76.92, 100.00)	−0.02	0.16 (0.00, 0.43)	0.50 (0.36, 0.69)	0.34 (0.49, 0.52)	0.367
	SVM_accel	69.00 (47.08, 72.22)	1.20 (0.00, 20.00)	95.08 (65.19, 100)	−0.04	0.04 (0.00, 0.26)	0.53 (0.36, 0.68)	2.92 (0.51, 0.54)	0.002
	CNN_gyro	60.46 (44.44, 72.16)	42.35 (0.00, 83.5)	67.42 (37.11, 100)	0.07 (−0.37, 0.42)	0.41 (0.00, 0.69)	0.56 (0.33, 0.74)	4.27 (0.54, 0.58)	<0.001
	CNN_accel	54.71 (27.78, 72.22)	28.61 (0.00, 100)	64.74 (19.61, 84.61)	−0.06 ^1^	0.26 (0.00, 0.63)	0.46 (0.16, 0.81)	−2.13 (0.43, 0.49)	0.983
Right	SVM_gyro	71.78 (66.67, 72.22)	1.40 (0.00, 20.00)	98.77 (88.27, 100)	0.00 (−0.08, 0.12)	0.11 (0.00, 0.31)	0.49 (0.37, 0.60)	−0.25 (0.49, 0.51)	0.599
	SVM_accel	70.50 (61.11, 72.22)	0.60 (0.00, 10.50)	97.38 (84.61, 100)	−0.02 ^1^	0.12 (0.00, 0.29)	0.49 (0.31, 0.66)	−0.12 (0.48, 0.51)	0.548
	CNN_gyro	50.38 (26.11, 72.22)	44.00 (0.00, 100)	54.10 (0.00, 88.84)	−0.12 ^1^	0.52 (0.00, 1)	0.48 (0.26, 0.68)	−1.83 (0.45, 0.50)	0.966
	CNN_accel	49.72 (27.78, 72.22)	43.05 (0.00, 100)	52.11 (0.00, 84.61)	−0.14 ^1^	0.32 (0.00, 0.53)	0.46 (0.18, 0.68)	−2.48 (0.43, 0.49)	0.993
Left	SVM_gyro	71.61 (66.67, 72.22)	0.00 (0.00, 0.00)	99.15 (92.31, 100)	−0.01 ^1^	0.00 (0.00, 0.00)	0.49 (0.34, 0.66)	−0.82 (0.48, 0.51)	0.794
	SVM_accel	71.06 (61.11, 72.22)	1.00 (0.00, 20.00)	98.00 (84.62, 100.00)	−0.01 ^1^	0.21 (0.00, 0.33)	0.51 (0.37, 0.62)	1.37 (0.49, 0.51)	0.085
	CNN_gyro	47.15 (27.78, 77.78)	64.32 (0.00, 100)	40.54 (0.00, 84.61)	0.05 (−0.27, 0.45)	0.54 (0.32, 0.81)	0.41 (0, 0.70)	3.06 (0.51, 0.57)	<0.001
	CNN_accel	49.91 (27.78, 72.22)	38.02 (0.00, 100)	54.48 (3.84, 84.61)	−0.08 ^1^	0.28 (0.00, 0.62)	0.44 (0.19, 0.70)	−3.95 (0.41, 0.46)	>0.999

^1^ Although the range of J is in [−1, 1], there is no practical interpretation of its negative values. Therefore, for the negative mean J index, the confidence interval is not reported.

## Data Availability

The clinical data can be obtained by contacting Gerald J. Jogerst at gerald-jogerst@uiowa.edu.

## Appendix A

Table A1 explains each clinical assessment tool’s functions, the measured variable, and the average required time to complete the test.

**Table A1:** An overview of fall-risk assessment clinical tests.

Tests	Function	Measurement	Assessment	Average Completion Time
FSST	Stepping over multiple low objects in different directions.	completion time	dynamic standing stability	<5 m
TUG test	Standing up from a chair, walking for three meters, turning, walking back to the chair, and sitting down.	completion time	gait and balance	<2 m
FRT	Measures the maximum forward reach without moving the feet (while standing in a fixed position).	maximum forward reach	stability and balance	<2 m
Step test	Stepping on the same foot on a stair without moving the other foot for 15 s.	number of steps	dynamic standing stability	<1 m
BBS	Performing 14 static and dynamic balance-related tasks, including standing, sitting, turning, reaching forward.	a total score of all the tasks	stability and balance	>15 m
4-stage balance	Standing in 4 different foot positions, in each stage, not moving the feet while keeping the balance.	total time of keeping the balance	stability and balance	<2 m
30 sec stand	Standing up from a chair and sitting back, repeating this move for 30 s.	number of stands, age- and gender-dependent	functional lower extremity strength	<2 m

Overall, some critical attributes and motion tasks are considered to find the most suitable test to further sensor measurement. Due to the real-world application of these tests and the limited time in clinical settings, the time required to complete the tests plays an important part in ranking the tests. The other key factor in choosing the optimized test is the ease of performance for both the clinician and the patients. Additionally, turning motion is critical to capture how subjects sway to keep their balance while turning and can be more critical than the gait motion in determining someone is at risk of falling.

Gait and dynamic stability are at the next level of importance and provide more fall-risk-related information than static stability and the rest of the features. Sitting and standing are also crucial because they would determine if someone could be independent in daily activities. The forward reach and stepping might not be as important as some of the other measures. Although stepping over an object is not asked in the TUG test, the clinician evaluates the patients’ step length and height to detect the risk of tripping over something or any correlated cognitive impairment. The reaching forward test challenges the patient’s balance and captures maintaining the balance at a two-foot stance. However, in the TUG test, the clinician evaluates the balance by watching the body’s center sway while getting off from the chair and turning.
